# Editorial

**Published:** 1894-07

**Authors:** 


### HOMŒOPATHIC PHYSICIAN,

A MONTHLY JOURNAL OF

HOMŒOPATHIC MATERIA MEDICA AND CLINICAL MEDICINE.

"If our school ever gives up the strict inductive method of Hahnemann, we are lost, and deserve only to be mentioned as a caricature in the history of medicine."—CONSTANTINE HERING.

Vol. XIV. JULY, 1894. No. 7.

### EDITORIAL.

CONSERVATISM IN MEDICINE.—Nothing in human character is more prominent than its conservatism.

Conservatism is that peculiar tendency of the mind to adhere to and maintain things as they exist. It is opposed to all change, all improvement, all progress. It sees no need of alternation of established order of human affairs, is but slightly impressed with the enormity of abuses, and will consent to no step that aims to reform such abuses.

As a consequence, progress of every kind is hindered, invention and discovery are discouraged, governmental reforms postponed, and every kind of advancement, improvement, and amelioration blocked.

It is the adamantine wall that must be thrown down or pierced by the discoverer, the inventor, the reformer, and the missionary.

Conservatism arises from a number of inherent causes.

To quote the editorial in this journal of April, 1893, referring to this subject:

"The human mind is so far the creature of habit, it is so deeply steeped in indolence, it is so incapable of investigation, it is so troubled with timidity that it has an instinctive aversion to any new idea implying change and the abandonment of established routine. This constitutes the *littleness* of the human mind, and renders it more subservient to those natural laws which govern lesser organizations. The Darwinian law of gradual evolution which pervades the animal and vegetable kingdoms generally governs it almost to the exclusion of its Divine intelligence and judgment, so that whatever principle of progress or elevation is presented to it must have traveled step by step through the most painful and vexatious stages of delay before it will be accepted."

This principle of conservatism developed thus in individuals dominates whole communities. It has caused the countries of Europe to fall behind the United States of America in the scale of advancement. It has caused the Chinese and Japanese to stand absolutely still for many centuries, these countries representing the most extreme instance of conservatism known. It is the cause of the great political struggle in England to-day, and has had an immense effect upon American politics.

Under its influence the votary of any system of politics, religion, or medicine, is but lightly affected by, or may be absolutely blind to the most convincing testimony to the errors or corruptions of the cause he espouses. On the other hand, he is equally unimpressed by the evidence of the truthfulness of the position taken by the opposite party, and so he does not investigate, and therefore does not change his views.

In the three greatest subjects of human interest, religion, politics, and medicine we see the foregoing observations most forcibly exhibited.

Conservatism thus constitutes one of the obstacles to the acceptance of Homœopathy. Adherents of the opposite school obeying the influences above noticed, neglect to inform themselves upon the subject and persistently denounce the system as false. That their information is not accurate is testified by the errors they make in stating the question.

In the April number of this journal, the effects of the habit of Generalization upon the acceptance of homœopathic doctrine were pointed out. In the June number Idealism was considered. In the present number we have treated of Conservatism. Thus there are three characteristic conditions of thought—

Generalization, Idealism, and Conservatism producing their effects upon the progress of the new medical treatment. An intelligent understanding of them enlightens us as to the cause of the opposition to our system of treatment.

Before us is a train of intellectual phenomena.

A system of medicine with grave defects and alarming incapacity to help the sick:

Another system of medicine with vast advantages over the preceding in the matter of alleviating suffering, but with a mechanism of treatment of surprising character, entirely outside of previous experience:

A cloud of witnesses and apostles of the latter testifying to its efficacy and therefore devoted adherents of the cause:

A powerful array of opponents admitting nothing, denying everything, and aggressive in their efforts to stamp out the system.

Ordinarily in accounting for these phenomena we say the opposition are inspired by scepticism, intolerance, jealousy, and indifference to truth.

This assertion does not give us a rational explanation of the facts. We need to look more closely at the workings of the human mind, and then we will find a series of phenomena such as have been explained in the editorials of three months past, which, sufficiently interesting in themselves, clear our minds as to the reasonableness or unreasonableness of the opposition to our school and teach us that this opposition is the most natural thing in the world. Then we are more content to endure it and remain quiet under the expectation of a future in which a higher personal education shall dissolve away these modes of thought like the mists of the morning.

---

NOTICE.—The editor regrets that, owing to the increased demands of his profession, and the amount of time required for the preparation of each number of the journal, the correspondence of the subscribers has been neglected. He desires to say that all letters will be answered as soon as leisure can be secured.

